# Identification of left atrial wall thickness in substrate mapping of atrial fibrillation

**DOI:** 10.3389/fcvm.2025.1592532

**Published:** 2025-06-25

**Authors:** Wei Liu, Shijie Li, Lina Dou, Chunai Hu, Bing Han

**Affiliations:** ^1^ Department of Cardiology, Xuzhou Central Hospital (Southeast University Affiliated Hospital), Xuzhou, Jiangsu, China; ^2^ Department of Cardiology, Peixian People’s Hospital, Xuzhou, Jiangsu, China; ^3^ Department of Radiology, Xuzhou Central Hospital (Southeast University Affiliated Hospital), Xuzhou, Jiangsu, China

**Keywords:** low voltage zone, delayed gadolinium-enhanced magnetic resonance imaging, left atrial wall thickness, voltage mapping, atrial fibrillation

## Abstract

**Objective:**

The objective of this study was to assess the clinical relevance of left atrial wall thickness (LAWT) in identifying electrophysiological substrate abnormalities.

**Methods:**

Eighty-two patients with atrial fibrillation undergoing first-time catheter ablation at Xuzhou Central Hospital between March 2016 and May 2023 were enrolled in this study. The left atrium was anatomically segmented into five regions, with all patients undergoing delayed gadolinium-enhanced magnetic resonance imaging (LGE-MRI) for quantitative assessment of parameters including left atrial wall thickness (LAWT, epicardial fat excluded). Bipolar voltage mapping was systematically performed to delineate low-voltage zones (LVZs) and calculate their relative area proportion within the total atrial surface for each patient. The regional segmentation method for left atrial voltage mapping was consistent with that used in late gadolinium-enhanced magnetic resonance imaging (LGE-MRI). Univariate and multivariate logistic regression analyses were conducted to identify clinical factors associated with LVZ formation. Receiver operating characteristic (ROC) curve analysis was employed to determine the optimal LAWT cutoff value for LVZ prediction, along with its corresponding sensitivity and specificity. Additionally, regional comparative analyses were performed between LGE-MRI-derived wall thickness measurements and their corresponding low-voltage zones identified by three-dimensional electroanatomic mapping.

**Results:**

The study cohort comprised 82 atrial fibrillation patients (44 paroxysmal AF, 38 persistent AF). Mean LAWT significantly differed between paroxysmal and persistent AF groups (2.6 ± 0.5 mm vs. 2.3 ± 0.4 mm, *P* = 0.02). Multivariate analysis identified age (OR = 1.111, 95% CI:1.03–1.19, *P* = 0.007), left atrial volume (OR = 1.029, 95% CI:1.003–1.055, *P* = 0.026), and LAWT (OR = 0.044, 95% CI:0.007–0.272, *P* = 0.001) as independent predictors of LVZs. Regional analysis revealed the septal wall was thinnest (1.76 ± 0.9 mm), followed by posterior (1.95 ± 0.4 mm) and bottom walls (2.62 ± 0.6 mm), with roof (2.89 ± 0.5 mm) and anterior walls (3.0 ± 0.4 mm) being thickest. Correspondingly, septal LVZ area was most extensive (22.5 ± 10.2%), exceeding posterior (15.3 ± 10.6%), bottom (12.6 ± 12.0%), roof (11.8 ± 10.0%), and anterior walls (10.8 ± 12.1%). ROC analysis demonstrated LAWT ≤ 2.3 mm predicted LVZs with 71% sensitivity and 68.2% specificity (AUC = 0.723, *P* < 0.001). Additional predictors included age >64.5 years (AUC = 0.722, sensitivity 65.9%, specificity 73.7%) and left atrial volume >119.2 ml (AUC = 0.682, sensitivity 61.4%, specificity 78.9%).

**Conclusions:**

This study demonstrate that LAWT significantly correlate with both atrial fibrillation progression and electroanatomical remodeling. Notably, regions exhibiting LAWT ≤ 2.3 mm predict more extensive LVZs. Our findings suggest that non-invasive LGE-MRI-based measurement of LAWT may enhance the detection rate of left atrial pathological substrates.

## 1 Introduction

Substrate mapping and modification have become essential components of radiofrequency ablation for atrial fibrillation (AF) (1). While low-voltage zones (LVZs) are generally accepted as predictor of localized myocardial fibrosis, they do not consistently correlate with the extent of atrial fibrosis (2). Recent studies have identified left atrial wall thickness (LAWT) and volume as additional predictors of pathological atrial remodeling in AF, though their relationship with LVZs distribution remains uncharacterized. Their impacts on LVZs mapping and the interrelationships between them have not been evaluated (3–10).

In this study, we integrated preprocedural delayed gadolinium-enhanced magnetic resonance imaging (LGE-MRI) assessment of LAWT and intraoperative three-dimensional electroanatomic mapping to systematically evaluate: (1) the association between structural remodeling (LAWT/volume) and electrophysiological substrate (LVZs), and (2) the potential for combined imaging-electrophysiological parameters to enhance the detection rate of left atrial pathological substrates.

## 2 Methods

### 2.1 Research population

**Figure 1:**
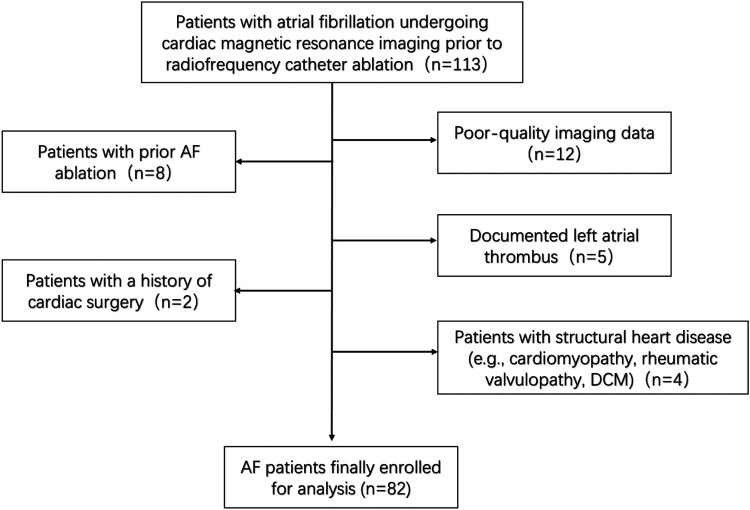
Flowchart.

With ethical approval from Xuzhou Central Hospital, this retrospective study analyzed patient data extracted from the hospital database on July 1, 2023, aiming to explore the association between left atrial wall thickness and fibrosis in atrial fibrillation patients. This study retrospectively enrolled 113 consecutive patients who underwent voltage mapping and LGE-MRI prior to radiofrequency ablation at Xuzhou Central Hospital (Xuzhou, China) between March 2016 and May 2023. The duration of AF was classified as either paroxysmal AF (less than 7 days) or persistent AF (more than 7 days). Persistent atrial fibrillation is defined as atrial fibrillation lasting from 7 days to 12 months, whereas long-standing persistent atrial fibrillation refers to guidelines where the duration exceeds 12 months. We excluded patients with previous catheter ablation, poor LGE-MRI image quality, documented left atrial thrombus, previous cardiac surgery, and structural heart disease. After applying the exclusion criteria, 82 patients with atrial fibrillation were ultimately included in the final analysis (Figure 1 Flowchart). Clinical characteristics were obtained from the medical record system. This study was conducted in accordance with the Declaration of Helsinki and received approval from the Research Ethics Committee of Xuzhou Central Hospital (Approval No. XZXY-LK-20230626-092) and the local National Health Service Research Ethics Committee (Study ID: MR-32-23-020405; www.medicalresearch.org.cn). Written informed consent was obtained from the patient before performing catheter ablation.

### 2.2 Late-gadolinium enhancement magnetic resonance imaging and analysis

**Figure 2:**
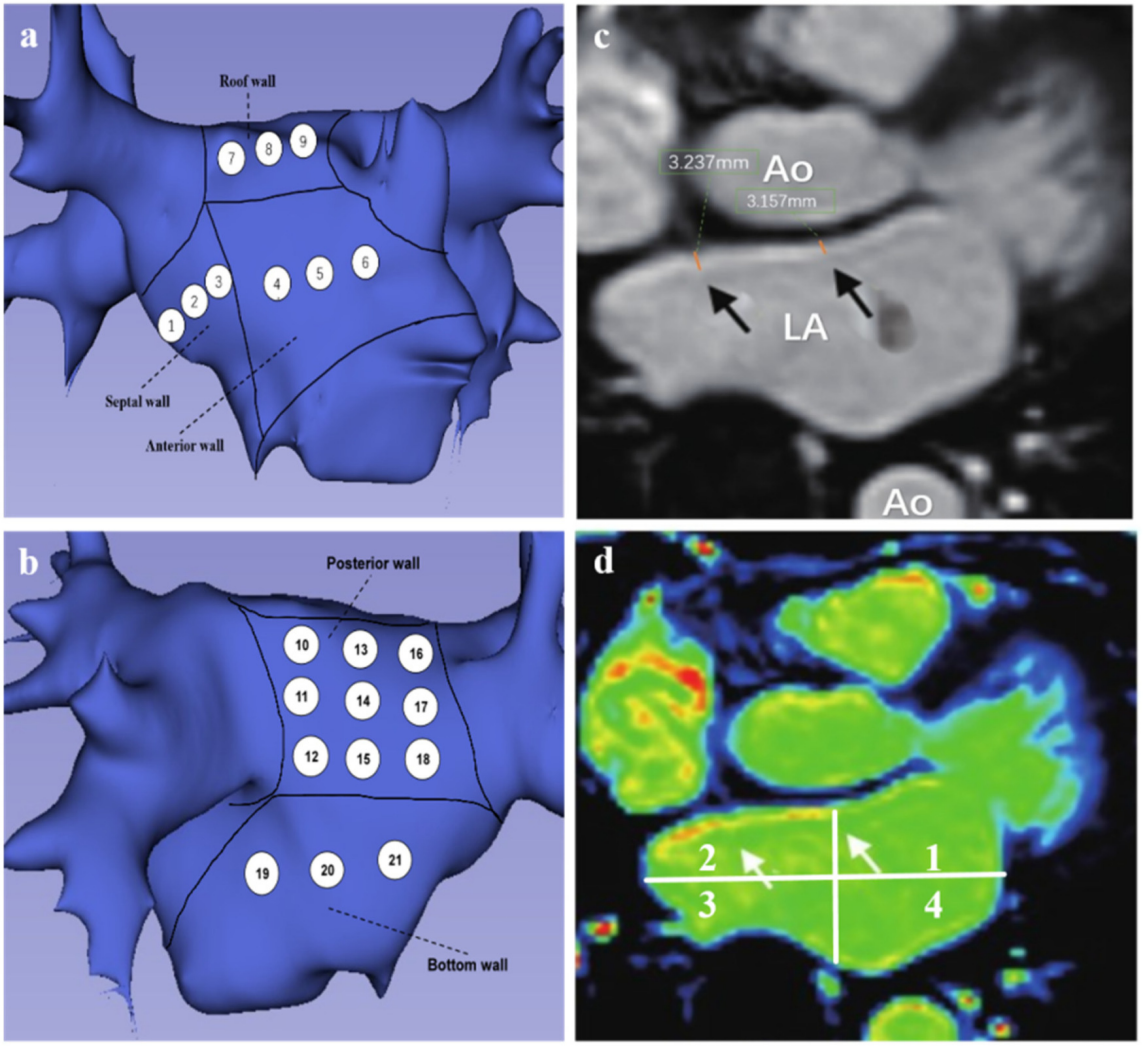
**(a,b)** Patterns of left atrial segment measurements. **(a)** Left atrial anteroposterior position, 1–3 represents the region of interstitial wall thickness measurement, 4–6 represents the region of AWT measurement, and 7–9 represents the region of RWT measurement. **(b)** Posterior-anterior position, 10–18 represents the PWT measurement region, 19–21 represents the BWT measurement region. **(c,d)** Representative LGE-MRI images of the left atrium. **(c)** Black arrows are examples of LAWT measurements. **(d)** magnetic resonance image of delayed enhancement zones (axial position), white arrows point to red and yellow zones representing fibrotic zones.

All patients underwent LGE-MRI imaging three days prior to the scheduled radiofrequency catheter ablation procedure. Examinations were performed using a 3.0 Tesla whole-body scanner (MAGNETOM Skyra, Siemens Healthineers, Erlangen, Germany) equipped with either a 5- or 32-element phased-array cardiac surface coil, with patients in the standard supine position. The imaging protocol included: (1)Localization sequences: TrueFISP (fast imaging with steady-state precession) for anatomical orientation of the left atrium. (2) Functional assessment: Balanced steady-state free precession (bSSFP) cine sequences for comprehensive evaluation of biventricular function (LV/RV). (3) Tissue characterization: T1-weighted 2D turbo fast low-angle shot (TurboFLASH) sequence for LGE-MRI. Cardiac synchronization was achieved through retrospective ECG gating to optimize phase selection during the cardiac cycle. For contrast-enhanced imaging, gadopentetate dimeglumine (Gd-DTPA, Magnevist®, Bayer AG, Leverkusen, Germany) was administered intravenously at standard dosing (0.1 mmol/kg body weight).The non-contrast localization scan was performed using a true fast imaging with steady-state precession (TrueFISP) gradient echo sequence to identify left atrial anatomy, with the following parameters: repetition time (TR) 221.90 ms, echo time (TE) 1.23 ms, slice thickness 4 mm, no gap, continuous acquisition, flip angle 60°, and field of view (FOV) 340 × 340 mm. For late gadolinium enhancement (LGE) imaging, a T1-weighted two-dimensional phase-sensitive inversion-recovery (PSIR) magnetization-prepared turbo fast low-angle shot (turbo-FLASH) sequence was employed with the following specifications: TR 700.00 ms, TE 1.09 ms, inversion time (TI) 300.00 ms, slice thickness 2 mm, 10% inter-slice overlap (acquiring 15–20 contiguous slices), flip angle 40°, and FOV 340 × 340 mm. All scans were obtained in the transverse plane with complete left atrial coverage, requiring an 8-second breath-hold per acquisition. All image acquisitions were performed at end-inspiration breath-hold. The total mean scanning time per patient was approximately 30 min. All cardiac MRI scans were acquired under sinus rhythm conditions to ensure optimal image quality. Images with signal-to-noise ratio (SNR) <2, calculated as the mean signal intensity of the left atrial wall divided by the standard deviation of background noise, were excluded to ensure analyzable tissue delineation. 3D multi-echo Dixon lipid-water separation sequence to assess epicardial fat; MR angiography was performed to assess PV anatomy; late gadolinium enhancement was performed to assess fibrosis/scar formation. After scanning was completed, the imaging images were transferred to an image post-processing workstation (Syngo.via) to assess parameters such as left atrial diameter, ejection fraction, left atrial volume and LAWT, and epicardial fat. Epicardial fat was defined as the zone of high signal between the myocardium and pericardium. On the basis of MRI cine sequence images, we measured left epicardial fat from the mitral annulus to the pulmonary bifurcation. Fat zones were manually tracked on consecutive cross-sectional images with post-processing software and multiplied by layer thickness to calculate left atrial-epicardial fat volume, volume/left atrial volume ratio. The LA was divided into 21 regions, with the pulmonary vein openings located at the level where the pulmonary veins enter the left atrium and the pulmonary vein sinus orifices located 10 mm from the pulmonary vein openings. The thickness of the three segments (right, center, and left) of the body of the left atrium was measured in the oblique sagittal plane in the cross section between the bilateral pulmonary vein openings. The thickness of the septum was measured in the oblique sagittal plane in the right midline. Average values for each region were used in the analysis. We divided the left atrium into five segments: septum(SW), anterior(AW), roof(RW), posterior(PW), and bottom(BW) walls, and used its 21 measurement points as measurement zone points (Figures 2a,b). To aid in this process, the initial visualization used a volume drawing tool in 3D slicer. The degree of delayed enhancement of the left atrium was classified into four grades: red and yellow colors on the CMR image were the zones of delayed enhancement, and green colors were the normal zones and chambers (Figure 2d), and based on the transverse-axial image, the left atrium was divided into four quadrants by two vertical median lines, and if there was no delayed enhancement in any of the four quadrants, the delayed enhancement was determined to be grade 0. Delayed enhancement level 1 is defined as zones of delayed enhancement in 1 quadrant of 2 or more consecutive levels. Delayed enhancement level 2 is defined as zones of delayed enhancement in 2 quadrants of 2 or more consecutive levels. Delayed enhancement was defined as level 3 if there were zones of delayed enhancement in 2 or more consecutive levels in 3 or more quadrants.

To evaluate measurement reproducibility, inter- and intra-observer variability analyses were performed in a randomly selected subset of 10 patients by two independent investigators (L.D. and C.H.). The intraclass correlation coefficients (ICCs) demonstrated excellent agreement, with values of 0.88 (intra-observer) and 0.94 (inter-observer). Additionally, Bland-Altman analysis revealed minimal measurement bias, with mean differences of 0.05 ± 0.32 mm (intra-observer) and 0.18 ± 0.37 mm (inter-observer), indicating high reproducibility.

### 2.3 Voltage mapping

All patients underwent radiofrequency ablation within 24 h following the MRI examination. The CARTO 3 electroanatomical mapping system (Biosense Webster) was employed to construct left atrial voltage maps during sinus rhythm. A 7-French decapolar catheter was utilized for left atrial endocardial mapping. Catheter contact was verified through assessment of electrogram stability, distance to the atrial geometry, and fluoroscopic confirmation of catheter movement consistent with cardiac contours. The acquired signals were processed using a bandpass filter (30–300 Hz), with each mapping point classified according to its bipolar voltage characteristics. Following transseptal puncture, the mapping procedure involved: Precise reconstruction of the mitral annulus and Systematic catheter advancement to pulmonary vein ostia. Gradual catheter withdrawal with point-by-point sampling of the anterior and posterior left atrial walls to generate a three-dimensional anatomical model. For voltage analysis, we established the following definitions: Normal myocardium: Bipolar voltage amplitude >1.5 mV; Fibrotic tissue: Bipolar voltage <0.5 mV. Consistent with MRI segmentation protocol, the left atrium was divided into five anatomical segments (septal, anterior, roof, posterior, and inferior walls) for regional wall thickness and LVZ assessment. LVZ distribution was quantified according to the left atrial subdivision scheme illustrated in Figures 2a,b.

### 2.4 Statistical analysis

The expression for continuous variables was mean ± standard deviation (X ± S). The independent samples t test was used to examine the difference between the two measurement groups after the normality test. The count data were expressed as number or percentage of cases (*n*/%). The left atrial low-voltage zone is defined as the area of the left atrial low-voltage zone divided by the total left atrial surface area. Left atrial delayed enhancement zone was expressed as the ratio of the zone of delayed enhancement signal to the entire left atrial surface zone. Univariate and multivariate logistics regression analyses were used to identify factors associated with LVZ. Receiver operating characteristic (ROC) curves were used to determine the accuracy of LAWT in predicting LVZ. Statistical significance was set *P* < 0.05. The statistical analysis was performed using GraphPad PRISM (version 26, GraphPad Software Inc., San Diego, CA, USA) and SPSS (version 9, IBM Corp., Armonk, NY, USA) with a valid copyright license obtained for their usage.

## 3 Results

### 3.1 Patient information

Table 1 presents the baseline characteristics of atrial fibrillation patients in the study population. This study enrolled a total of 82 patients with a mean age of 63 ± 9 years, including 52 males (63%) and 30 females (37%). Among all participants, 44 cases (54%) were paroxysmal atrial fibrillation (PAF), 29 cases (35%) were persistent atrial fibrillation (PeAF), and 9 cases (11%) were long-standing PeAF.

**Table 1:** Characteristics of patients with atrial fibrillation.

Baseline characteristics of the study population	*N* = 82
Age (y)	63 ± 9
Gender (Male)	52 (63%)
PAF	44 (54%)
PeAF	29 (35%)
Long-standing PeAF	9 (11%)
Atrial fibrillation duration, months	12.5 (4.0–48)
Smoking	20 (24%)
Myocardial infarction	3 (3%)
HF	5 (6%)
Hypertension	43 (52%)
Diabetes mellitus	11 (13%)
Stroke	5 (6%)
Drugs	
warfarin	25 (30%)
amiodarone	40 (48%)
β-blocker	56 (68%)
New oral anticoagulants	57 (69%)
CHA2DS2-VASc score	1 (1–2)
BMI	25 ± 3.5
LVEF	56.5 (55,58)

PAF, paroxysmal atrial fibrillation, PeAF, persistent atrial fibrillation; HF, heart failure; LVEF, left ventricular ejection fraction.

### 3.2 Patients MRI characteristics

The left atrial parameters measured by CMR are shown in Table 2. By comparing and analyzing the differences in the left atrial parameters measured by CMR between the two groups of patients with PAF and PeAF, we found that there were statistically significant differences in LAWT (2.6 ± 0.5 vs. 2.3 ± 0.4, *P* = 0.02). The left atrium was divided into five segments, and the wall thickness of each segments were measured. We found that SWT was higher in patients with PAF than in patients with PeAF (2.0 ± 0.9 vs. 1.6 ± 0.85, *p* < 0.001). AWT was higher in PAF than PeAF (3.0 ± 0.8 vs. 2.57 ± 0.6, *P* < 0.001). PWT was higher in patients with PAF than PeAF (2.1 ± 0.55 vs. 1.6 ± 0.4, *P* = 0.003). No significant differences were observed between the two groups in terms of RWT and BWT. There was no statistically significant difference in LVEF between the two groups (*P* = 0.209). In terms of left atrial volume, PeAF had a larger left atrial volume than PAF (119 ± 27 ml vs. 107 ± 24 ml, *P* = 0.047). No statistically significant differences were seen in left atrial epicardial fat volume and epicardial fat volume to atrial volume ratio. In the left atrial delayed intensification grading analysis, 25 patients (56%) with PAF had a delayed enhancement grading of 0, 9 patients (20%) with PAF had a delayed enhancement grading of 1, 5 patients (11%) with PAF had a delayed enhancement grading of 2, and 5 patients (11%) with PAF had a delayed enhancement grading of 3. In patients with PeAF, 13 (34%) had a delayed enhancement grading of 0, 6 (16%) had a delayed enhancement grading of 1, 13 (34%) had a delayed enhancement grading of 2, and 6 (16%) had a delayed enhancement grading of 3.

**Table 2:** Patient CMR characteristics.

Patient CMR characteristics	PAF (*n* = 44)	PeAF (*n* = 38)	*P* value
Mean LAWT(mm)	2.6 ± 0.5	2.3 ± 0.4	0.02
SWT (mm)	2.0 ± 0.9	1.6 ± 0.85	<0.001
AWT (mm)	3.0 ± 0.8	2.57 ± 0.6	<0.001
RWT (mm)	2.9 ± 0.5	2.84 ± 0.73	0.167
PWT (mm)	2.1 ± 0.55	1.6 ± 0.4	0.003
BWT (mm)	2.68 ± 0.6	2.6 ± 0.6	0.25
LVEF(%)	57.5 (55,58)	55 (53,58)	0.209
LA EAT(ml)	107 ± 24 ml	119 ± 27	0.047
LA EAT (ml)	53.5 ± 15.6	54 ± 18.3	0.597
LA volume index (ml/m^2^)	14.7 ± 5.3	15.2 ± 5.1	0.34
LA EAT/LA volume	0.29 ± 0.15	0.33 ± 0.14	0.25
Left atrial delayed enhancement grading			
0	25 (56%)	13 (34%)	0.041
1	9 (20%)	6 (16%)	0.586
2	5 (11%)	13 (34%)	0.013
3	5 (11%)	6 (16%)	0.558

LA, left atrium; EAT, epicardial fat; Septal wall thickness; AWT, anterior wall thickness; RWT, roof wall thickness; PWT, posterior wall thickness; BWT, bottom wall thickness; LVEF, left ventricular ejection fraction; LAWT, left atrial wall thickness.

### 3.3 Univariate and multivariate logistics regression analysis of clinical factors that can influence LVZ

The average number of mapping points per patient was 822 ± 330. According to the univariate logistics regression analysis (Table 3), The age(OR = 1.096, 95% CI:1.035–1.162, *P* = 0.002), gender(OR = 2.942, 95% CI: 1.134–7.634, *P* = 0.027), left atrial volume(OR = 1.974, 95% CI: 1.957–1.993, *P* = 0.006) and LAWT (OR = 0.629, 95% CI: 0.580–0.862, *P* = 0.003) were associated with LVZ (Table 4). Multivariate logistics regression analysis identified age (OR = 1.111, 95% CI: 1.03–1.19, *P* = 0.007), LA volume (OR = 1.029, 95% CI: 1.003–1.055, *P* = 0.026) and LAWT(OR = 0.044, 95% CI: 0.007–0.272, *P* = 0.001) as independent determinants of LVZ. In addition, gender, LA activation time and LVEF were not associated with LAWT (Table 3).

**Table 3:** Univariate and multivariate logistics regression analysis of clinical factors that can influence LVZ.

Clinical factors	Univariate analysis	*P* value	Multivariate analysis	*P* value
	*OR* (95% CI)		*OR* (95% CI)	
Age	1.096 (1.035–1.162)	0.002	1.111 (1.03–1.19)	0.007
Gender (female)	2.942 (1.134–7.634)	0.027	1.889 (0.503–7.099)	0.346
PeAF	1.962 (0.813–4.737)	0.134		
LVEF	1.004 (0.939–1.074)	0.896		
LA volume	1.974 (1.957–1.993)	0.006	1.029 (1.003–1.055)	0.026
LA activation time	0.997 (0.981–1.013)	0.720		
LAWT	0.629 (0.580–0.862)	0.003	0.044 (0.007–0.272)	0.001

PeAF, persistent atrial fibrillation; LVEF, left ventricular ejection fraction; LA, left atrium; LAWT, left atrial wall thickness.

**Table 4:** Regional analysis between LAWT and LVZs.

Regional descriptions of each LVZ and LAWT segment	SW	AW	RW	PW	BW	*P* value
LAWT (mm)	1.76 ± 0.9 *^,†,‡,¶^	3.0 ± 0.4^‡^^,^^¶^	2.89 ± 0.5^‡^	1.95 ± 0.4^¶^	2.62 ± 0.6	<0.001
LVZ (%)	22.5 ± 10.2**^,‡,¶^	15.3 ± 10.6	12.6 ± 12.0	11.8 ± 10	10.8 ± 12.1	<0.001

Date are mean ± SD. *P < 0.001 vs. AW. **P < 0.01 and ^†^*P* < 0.001 vs. RW. ^‡^*P* < 0.001 vs. PW. ^¶^*P* < 0.001 vs. BW. SW, septal wall; AW, anterior wall; RW, roof wall; PW, posterior wall; BW, bottom wall; LAWT, left atrial wall thickness.

### 3.4 Descriptive characterization of the distribution of CMR-measured wall thicknesses of LA segments and the LVZ of the corresponding CARTO three-dimensional electroanatomical mapping of LA

In the regional LA analysis (Table 4), the thickness of SW was thinner than the thickness of AW, RW, PW, BW (1.76 ± 0.9, 1.95 ± 0.4, 2.62 ± 0.6, 2.89 ± 0.5, 3.0 ± 0.4 mm). Consistent with a relationship between regional LAWT and regional LVZ, the LVZs of SW was more extensive than the LVZ of AW, RW, PW and BW (22.5% ± 10.2%, 15.3% ± 10.6%, 12.6% ± 12.0%, 11.8% ± 10%, 10.8% ± 12.1%).

### 3.5 Correlation analysis between LAWT and left atrial volume

**Figure 3:**
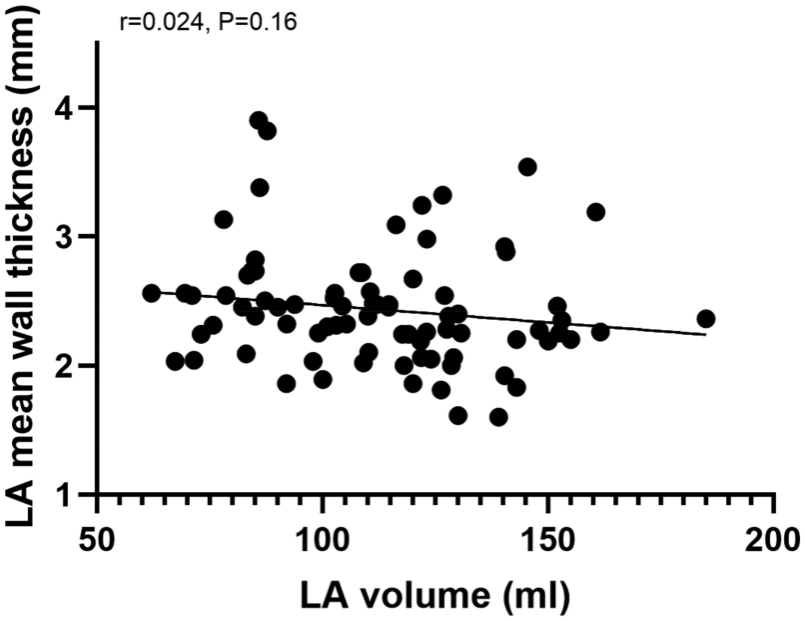
Correlation analysis of left atrial mean wall thickness and left atrial volume.

Correlation analysis of mean LAWT and left atrial volume, we found that the correlation between mean LAWT and left atrial volume was not significant (*r* = 0.024, *P* = 0.16), see Figure 3.

### 3.6 ROC curves to predict optimal age, left atrial volume and LAWT values for LVZ

**Figure 4:**
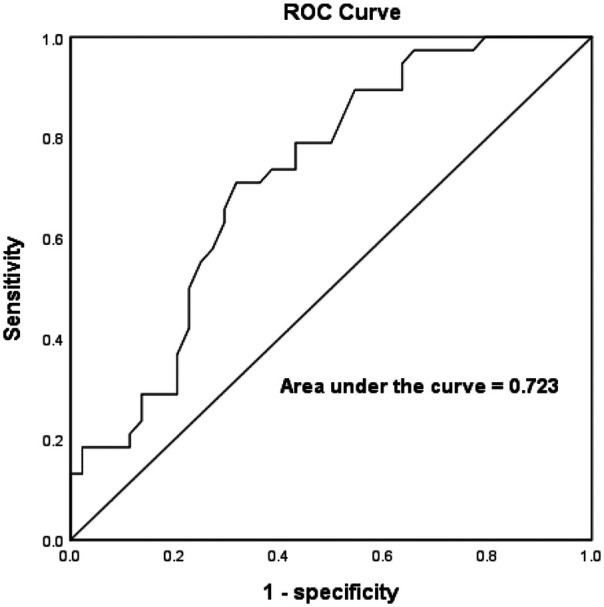
ROC curves for LAWT prediction of LVZ in AF.

The sensitivity and specificity of the best LAWT for predicting the presence of LVZ was analyzed by using the ROC curve to go for the prediction of LVZ. The results showed that LAWT better predicted the presence of LVZ with an optimal cutoff value of 2.3 mm, a sensitivity of 71.1%, a specificity of 68.2%, and an zone under the ROC curve (AUC) of 0.723 (*P* < 0.001, 95% CI:0.613–0.832), as shown in Figure 4.

**Figure 5:**
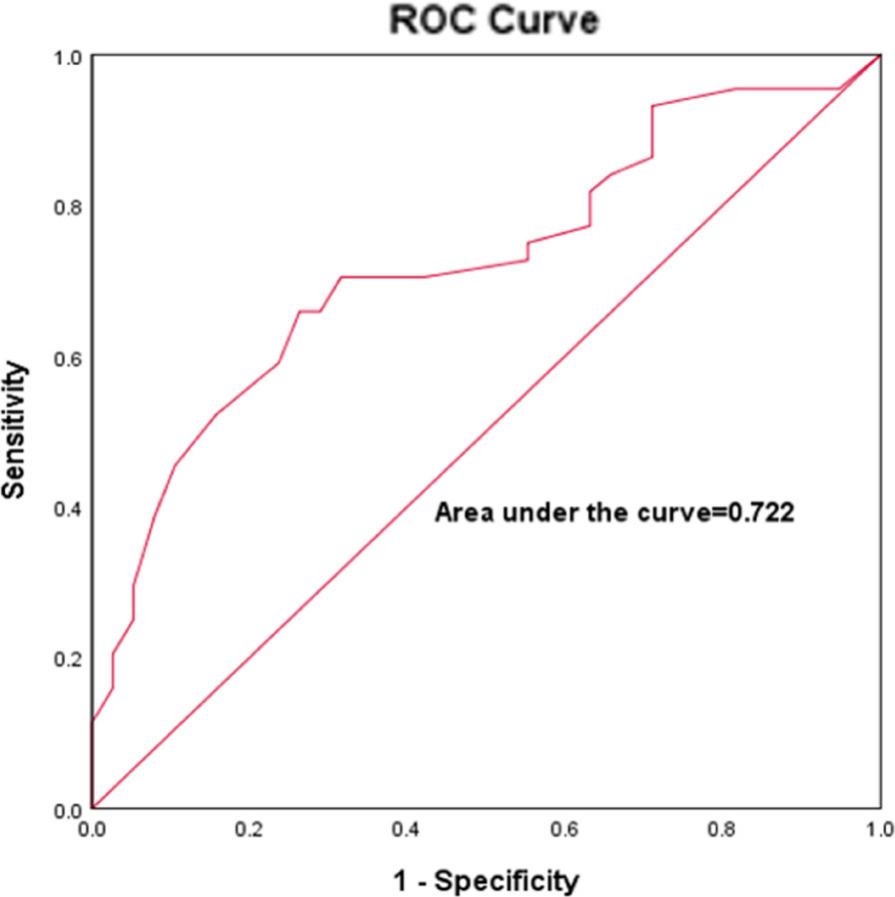
ROC curves for age predicted LVZ in AF.

The sensitivity and specificity of the optimal age for predicting LVZ was analyzed by using ROC curves to go through the best age for predicting LVZ. The results showed that the optimal age cut-off value for predicting LVZ was 64.5 years, with a sensitivity of 65.9% and a specificity of 73.7%, and an AUC:0.722 (*P* < 0.001, 95% CI:0.612–0.387), as shown in Figure 5.

**Figure 6:**
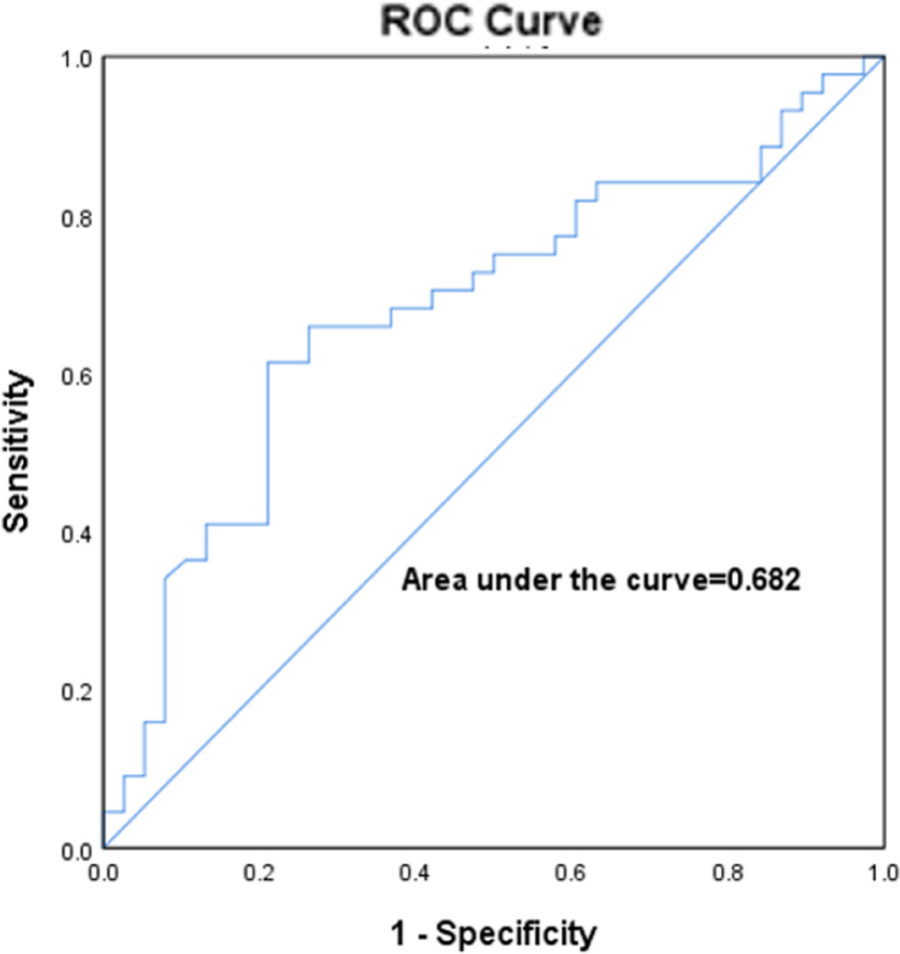
ROC curves for left atrial volume predicted LVZ in AF.

Figure 6 represents the ROC curve of left atrial volume for predicting the LVA, with an optimal cut-off value of 119.2 ml and a sensitivity of 61.4%; the specificity was 78.9%, AUC:0.682 (*p* = 0.005, 95% CI:0.564–0.799).

## 4 Discussion

The technology for measuring LAWT using CT has now been well established. The following advantages of CT imaging have been demonstrated: High imaging resolution and rapid acquisition capability are provided. Widespread clinical adoption and straightforward operation have been achieved. Simultaneous assessment of left atrial appendage thrombus can be performed. In recent years, CT-based measurement of LAWT has been widely adopted in atrial fibrillation ablation studies. Different radiofrequency ablation energy levels have been selected based on regional variations in atrial wall thickness. By integrating preoperative LAWT measurements with intraoperative voltage mapping, personalized ablation strategies have been successfully guided. Recent studies have demonstrated that the LAWT-guided individualized ablation index strategy achieves comparable clinical outcomes with reduced radiofrequency energy delivery compared to conventional non-personalized approaches (11–14). Previous studies have predominantly focused on measuring wall thickness at the pulmonary vein ostia (both left and right), whereas the present investigation specifically targets the atrial body region for wall thickness quantification. Falasconi et al. concluded from CT imaging scans of the wall thickness of the anterior wall of the left pulmonary vein that it was 2.04 (2.0–2.4) mm in patients with paroxysmal atrial fibrillation and 1.98 (1.8–2.2) mm in persistent atrial fibrillation (11). Alderete et al. reported anterior pulmonary vein wall thickness of 1.55 (1.38–1.95)mm in patients with paroxysmal atrial fibrillation scanned by CT imaging. The posterior wall thickness of the pulmonary veins was 1.16 (0.94–1.28) mm (12). Teres et al. found that in patients with paroxysmal atrial fibrillation the thickness of the anterior wall of the pulmonary veins was 1.28 ± 0.41 mm and the thickness of the posterior wall of the pulmonary veins was 0.99 ± 0.30 mm (13). Nakamura et al. investigated the interaction of atrial wall thickness and lesion substrate with AF rotors by performing delayed-enhancement magnetic resonance scans in patients with atrial fibrillation (atrial: 2.3 ± 0.3 mm, bottom: 2.3 ± 0.2 mm, LAA base: 2.2 ± 0.2 mm, lateral: 2.2 ± 0.2 mm, posterior: 2.0 ± 0.4 mm, PV antrum: 2.3 ± 0.3 mm, roof: 2.4 ± 0.4 mm, and septum: 2.1 ± 0.2 mm. posterior: 2.0 ± 0.4 mm, PV antrum: 2.3 ± 0.3 mm, roof: 2.4 ± 0.4 mm, and septum: 2.1 ± 0.2 mm, *p* = 0.094) (15). We found variations in atrial wall thickness across different studies, which may be attributed to differences in the included populations, imaging modalities, and parameters. Comparison of the two techniques for measuring left atrial wall thickness revealed slightly higher values in our study compared to CT imaging (16). This discrepancy may be primarily attributed to MRI partial volume effects, blurred fibrotic boundaries, MRI's greater sensitivity in detecting trabeculations, and CT's superior resolution in thin regions. This study utilized LGE-MRI to assess the left atrium. Unlike CT imaging, LGE-MRI enables detection of atrial fibrosis extent. Current evidence suggests certain limitations in electroanatomical mapping. Preprocedural LGE-MRI quantification of myocardial fibrosis helps overcome bipolar voltage mapping inaccuracies caused by regional wall thickness variations, thereby improving the detection rate of arrhythmogenic substrates in atrial fibrillation. Moreover, LGE-MRI is radiation-free and can differentiate epicardial fat from myocardium, providing more accurate measurements of left atrial wall thickness (17–19). However, compared to CT, the current LGE-MRI technique still has several limitations: longer scan times, poorer patient tolerance, and slightly lower spatial resolution.

Our findings demonstrate a statistically significant difference in mean LAWT between paroxysmal and persistent AF patients. Consistent with prior reports (8), persistent AF patients exhibited thinner LAWT compared to paroxysmal AF cases. Nakamura et al. (8) observed progressive atrial wall remodeling during the transition from paroxysmal to chronic AF. Notably, paroxysmal AF patients showed significantly greater anterior wall thickness compared to both AF-naïve patients and non-AF controls (2.4 ± 0.2 mm vs. 1.9 ± 0.2 mm vs. 2.1 ± 0.2 mm, respectively). This CT-based study suggests dynamic LAWT variations across AF subtypes, with progressive anterior wall thinning accompanying AF progression, reflecting complex anatomical remodeling processes associated with atrial dilatation. The CT-measured LAWT values were systematically lower than ex vivo tissue measurements, potentially attributable to differential loading conditions *in vivo* vs. ex vivo, or possibly indicating inherent CT underestimation of true wall thickness. Histological studies provide complementary insights: Morillo et al. (20)documented rapid atrial pacing-induced ultrastructural changes in canine models, including mitochondrial enlargement and sarcoplasmic reticulum disruption after 6 weeks. Ausma et al. (21) further characterized atrial cardiomyocyte hypertrophy in goat models of sustained AF, associated with myolysis and perinuclear glycogen accumulation. Human histological studies consistently demonstrate atrial myocyte hypertrophy and interstitial fibrosis in AF patients, corroborating these experimental findings.

Previous studies have consistently demonstrated that LVZ identified by electroanatomic mapping exhibit strong concordance with LGE regions on LGE-MRI, as both modalities reflect the underlying arrhythmogenic substrate of atrial fibrillation - specifically, myocardial fibrosis (2, 22). Importantly, LGE-CMR provides superior capability for quantifying the extent of myocardial fibrosis compared to electrophysiological mapping techniques. Myocardial fibrosis has been well-established as playing a pivotal role in the pathogenesis of atrial fibrillation (23, 24). Our study proposes several pathophysiological mechanisms that may explain the correlation between LAWT and LVZ (1): Hemodynamic Remodeling: LAWT alterations likely reflect early-stage structural remodeling in AF, preceding detectable atrial dilation. The left atrium, being a thin-walled chamber, demonstrates marked sensitivity to volume overload. The characteristic hemodynamic changes in AF—including impaired ejection fraction and elevated atrial pressure/volume—potentially lead to wall thinning through sustained mechanical stretch and compressive forces. (2) Histopathological Basis: The structural remodeling process encompasses both cardiomyocyte apoptosis and interstitial fibrosis, which collectively contribute to LAWT reduction. Furthermore, the rapid atrial rate characteristic of AF may cause relative myocardial ischemia and oxidative stress, thereby accelerating myocyte apoptosis (24). This pathological cascade results in progressive voltage reduction, ultimately facilitating LVZ formation. (3) Anatomic Considerations: The observed predominance of anterior wall LVZ may be attributable to mechanical interactions with contiguous anatomical structures. In particular, persistent compression from the adjacent ascending aorta could induce anterior wall remodeling, promote fibrotic changes, and consequently lead to voltage reduction in this region. (4) Technical limitations of voltage mapping: Current bipolar voltage mapping has inherent detection limitations. Thicker myocardial regions may harbor mid-myocardial or epicardial fibrosis undetectable by endocardial mapping. While endocardial fibrosis can be reliably identified, the accurate detection of mid-wall and epicardial low-voltage zones remains challenging. Current literature highlights significant limitations in three-dimensional mapping systems for detecting scar tissue in thick-walled myocardial regions, as demonstrated by Selcuk et al. (25) in animal studies. While our study confirms an independent correlation between LAWT and LVZ, it is crucial to acknowledge potential systematic biases introduced by technical limitations of bipolar voltage mapping. The amplitude of bipolar electrograms exhibits a strong dependence on myocardial thickness, following an exponential decay pattern. In regions with thicker atrial walls, mapping catheters may only capture electrical activity from the endocardial 40%–60% of the myocardial wall. This “distance-dependent signal attenuation phenomenon” can lead to two distinct diagnostic errors: False-negative fibrosis detection: When fibrotic changes are confined to mid-myocardial or epicardial layers (characteristic of early AF remodeling), endocardial bipolar voltages may remain within normal ranges (>0.5 mV). False-positive LVZ identification: Anatomically thick but healthy myocardial regions may be misclassified as LVZs due to signal attenuation. This effect is particularly pronounced in high-impedance patients (e.g., those with BMI > 30 showing 23% greater signal attenuation, *p* < 0.01).

In this study, we found no correlation between LAWT and left atrial volume, and there is a current controversy regarding the relationship between atrial volume, LAWT, and myocardial fibrosis. Yosuke et al (26) found no correlation between LAWT and left atrial volume and deduced that the hypothesis of thinning of the left atrial wall caused by pressure overload of the left atrial volume was not valid. Thinning of the LAWT is most likely due to atrial remodeling and does not correlate with volume. Floria (27) concluded that the relationship between left atrial volume and left atrial fibrosis is quite unpredictable because some patients have extensive fibrosis but normal left atrial volume.

Multiple clinical studies and meta-analyses have demonstrated that ablation of LVZ significantly reduces AF recurrence rates of post-procedure. Our study identifies left atrial wall thickness (LAWT) as an independent predictor of LVZ, with a threshold value of ≤2.3 mm predicting more extensive low-voltage areas. This finding provides a novel non-invasive parameter for preoperative LVZ prediction. Furthermore, quantitative assessment of LAWT and related parameters through preprocedural LGE-CMR imaging can effectively predict LVZ severity. The integration of these preoperative imaging metrics with intraoperative mapping may overcome the inherent limitations of bipolar voltage mapping, thereby improving the detection rate of AF substrates and providing critical information for therapeutic decision-making in AF management.

There are several limitations of this study. First, this study is a single-center observational study. The sample size is small, and a large sample size study is needed for further validation. Second, the prevalence of LVZ in our study cohort was higher than reported in previous literature, which may be attributed to clinician referral patterns. This observation suggests potential patient selection bias in our study population. Third, using current magnetic resonance imaging techniques to diagnose atrial scarring has some limitations and does not validate histologic evidence well. In addition, the measured LAWT may be biased because the heart keeps beating and MRI cannot avoid motion artifacts.

## 5 Conclusions

LAWT is associated with AF progression and left atrial electroanatomic remodeling, and for regions with LAWT ≤ 2.3 mm predicts more extensive LVZ. Our findings suggest that non-invasive CMR-based measurement of left atrial wall thickness (LAWT) may enhance the detection rate of left atrial pathological substrates.

## Data Availability

The raw data supporting the conclusions of this article will be made available by the authors, without undue reservation.
